# Supplementary material for: Total escape of SARS-CoV-2 from dual monoclonal antibody therapy in an immunocompromised patient
Source: Nat Commun. 2023 Apr 10;14:1999. doi: 10.1038/s41467-023-37591-w (PMC10085998; doi:10.1038/s41467-023-37591-w)
Supplement: Supplementary file 1 — Supplementary Information [file 41467_2023_37591_MOESM1_ESM.pdf]

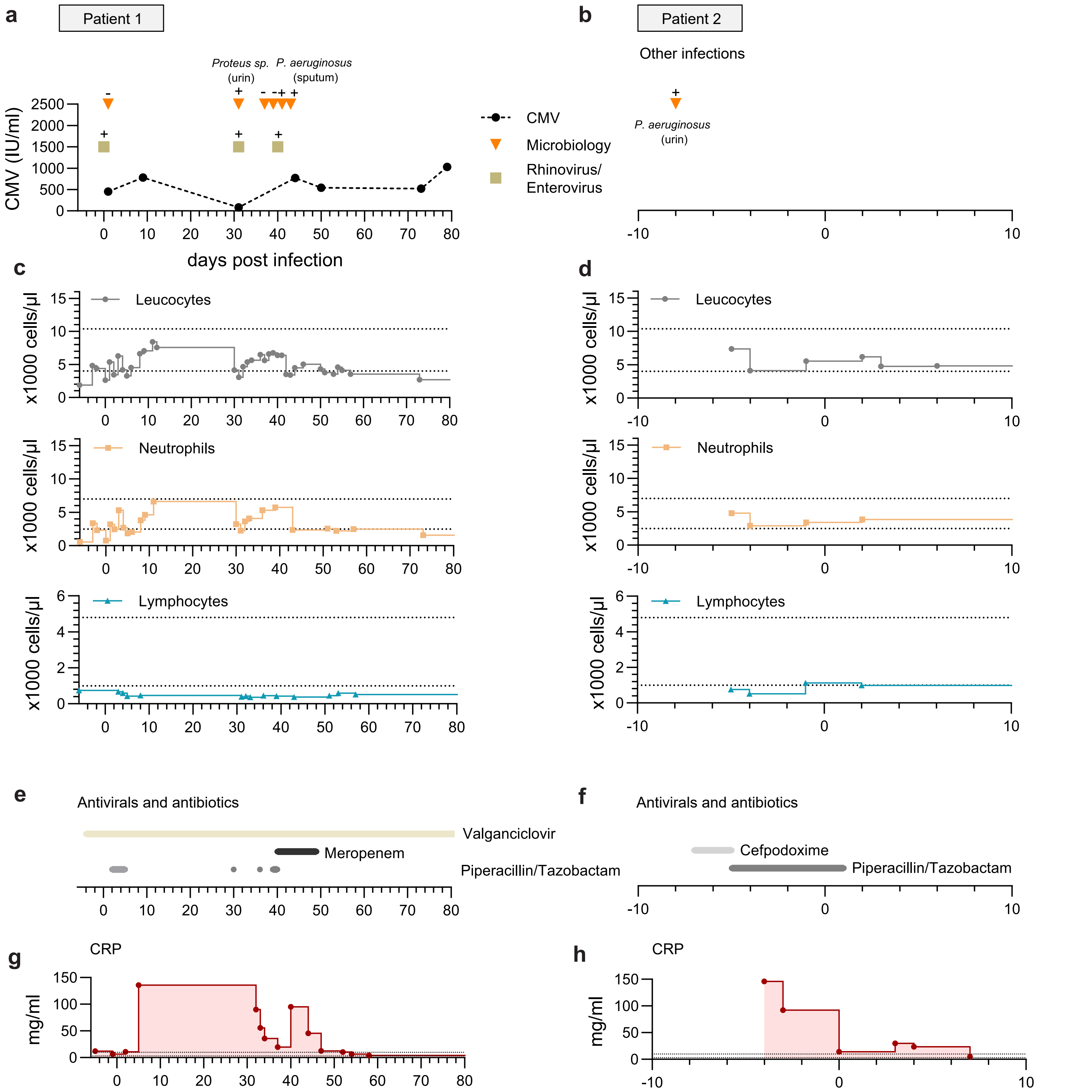

Supplementary figure 1. Temporal overview of clinical parameters of patient 1 and 2. Data for (a, c, e, g) patient 1 and (b, d, f, h) patient 2 in respect to the first positive SARS-CoV-2 qPCR result (d0). Timeline for (a, b) infections other than SARS-CoV-2, (c, d) Leucocyte, Neutrophils and Lymphocyte counts, (e, f) antiviral or antimicrobial treatment regimens and (g,h) of C reactive protein (CRP).

a

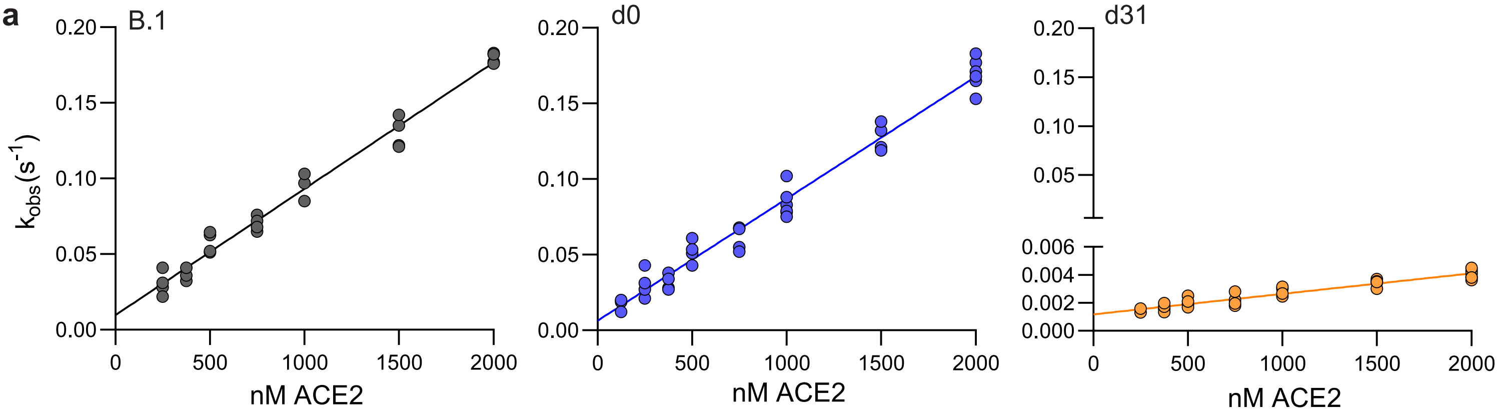

b

Spike variant	k_{on} [$mM^{-1}s^{-1}$]	k_{off} [s^{-1}]	K_D (kinetic) [nM]	k_{off} (diss. phase) [s^{-1}]	K_D (diss. phase) [nM]	K_D (amplitudes) [nM]
B.1	83.49 ± 2.41	0.0097 ± 0.0027	116 ± 32	0.0121 ± 0.0019	145 ± 23	181 ± 32
d0	80.81 ± 2,6	0.0061 ± 0.003	76 ± 37	0.00483 ± 0.001	60 ± 13	72 ± 16
d31	1.47 ± 0.09	0.0012 ± 0.0001	803 ± 89	0.0014 ± 0.0001	962 ± 112	770 ± 90

c

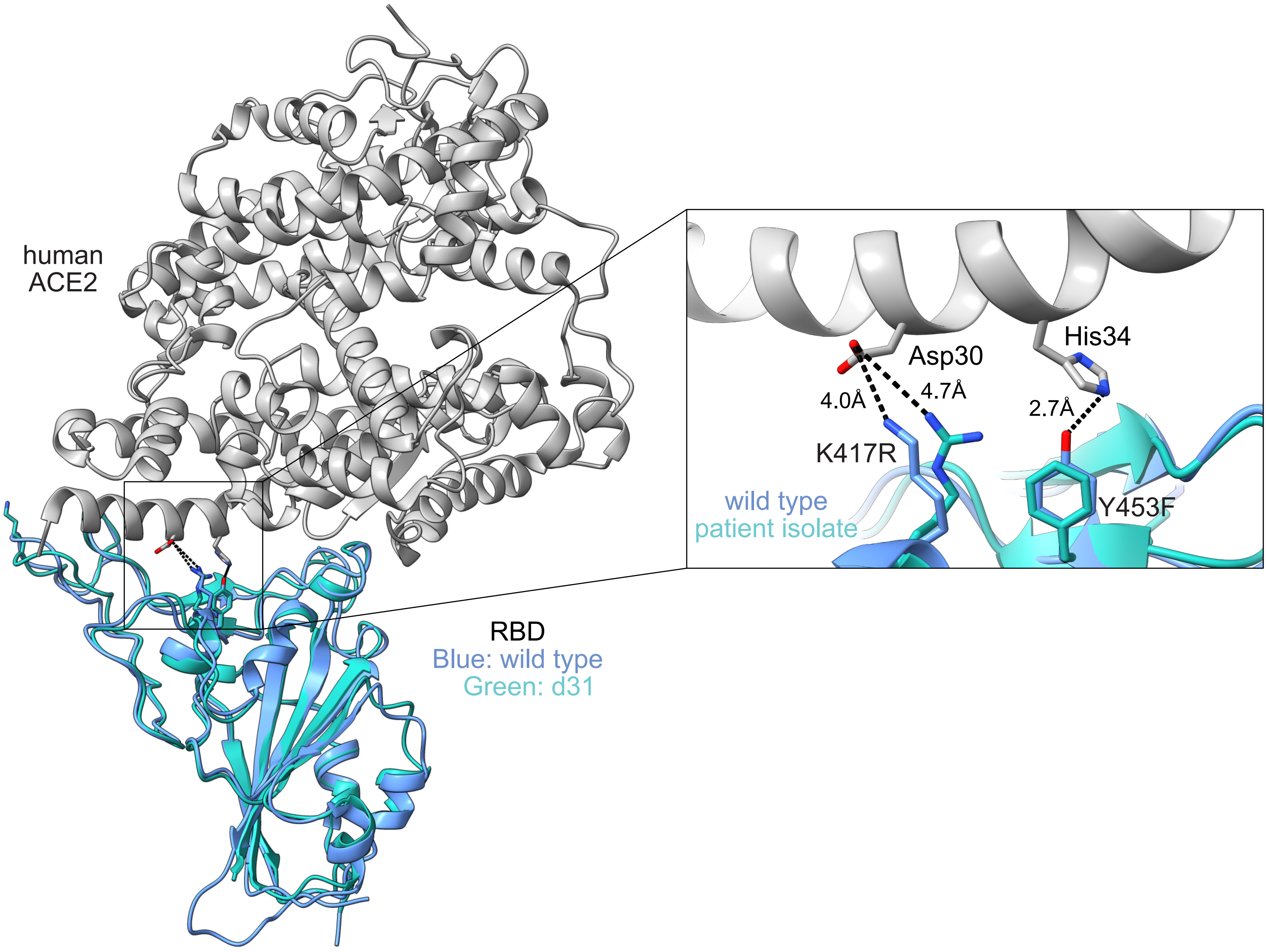

Supplementary figure 2. Biolayer interferometry binding measurements of ACE-2 binding to the immobilised S variants. (a) Dependence of the observed rate constant (k_{obs}) on ACE-2 concentration for the S of B.1, d0 and d31. (b) Thermodynamic parameters for ACE-2 binding to the S of B.1, d0 and d31. Association rate constants (k_{on}) were determined from the slopes of the linear regression in (a). Dissociation rate constants (k_{off}) were determined from the intercepts of the linear regression and through independent analysis of the dissociation phase (k_{off}(diss. phase)). The K_D values were calculated from the kinetic data as k_{off}/k_{on} (K_D (kinetic)), from k_{off}(diss. phase)/k_{on} (K_D (kinetic)) and from analysis of the dependence of fractional saturation on ACE-2 concentration shown in Fig. 2f (K_D(amplitudes)). Source data are provided in the source data file. (c) 3D presentation of the RBD of the SARS-CoV-2 spike protein and the d31 isolate RBD predicted by AlphaFold 2 (green) bound to ACE-2 (PDB accession number: 7u0n, blue).

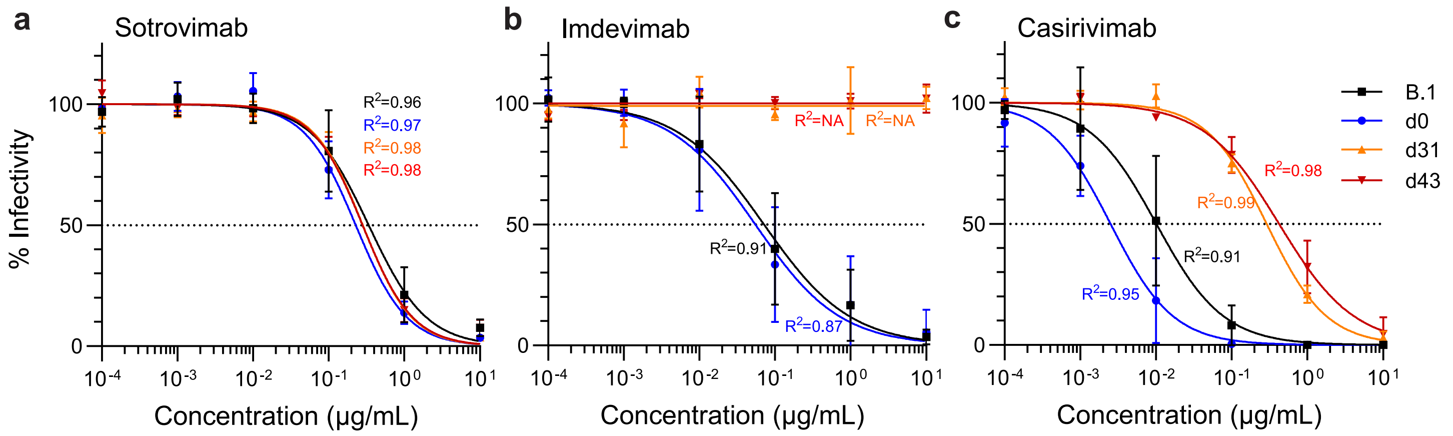

Supplementary figure 3. Curve fits for the neutralizing capacity of the mAbs. Serial 10-fold dilutions of the monoclonal antibodies (a) Sotrovimab, (b) Imdevimab and (c) Casirivimab were incubated with 100 pfu of the B.1 isolate or the three patient isolates (d0, d31, d43) and analysed by plaque assay. Plotted are the curve fits (mean ± SD) of the serial dilutions (n=3 independent experiments). The goodness of fit (R^2) is indicated at the respective curves. The dotted line marks 50 % infectivity. Source data are provided in the source data file.

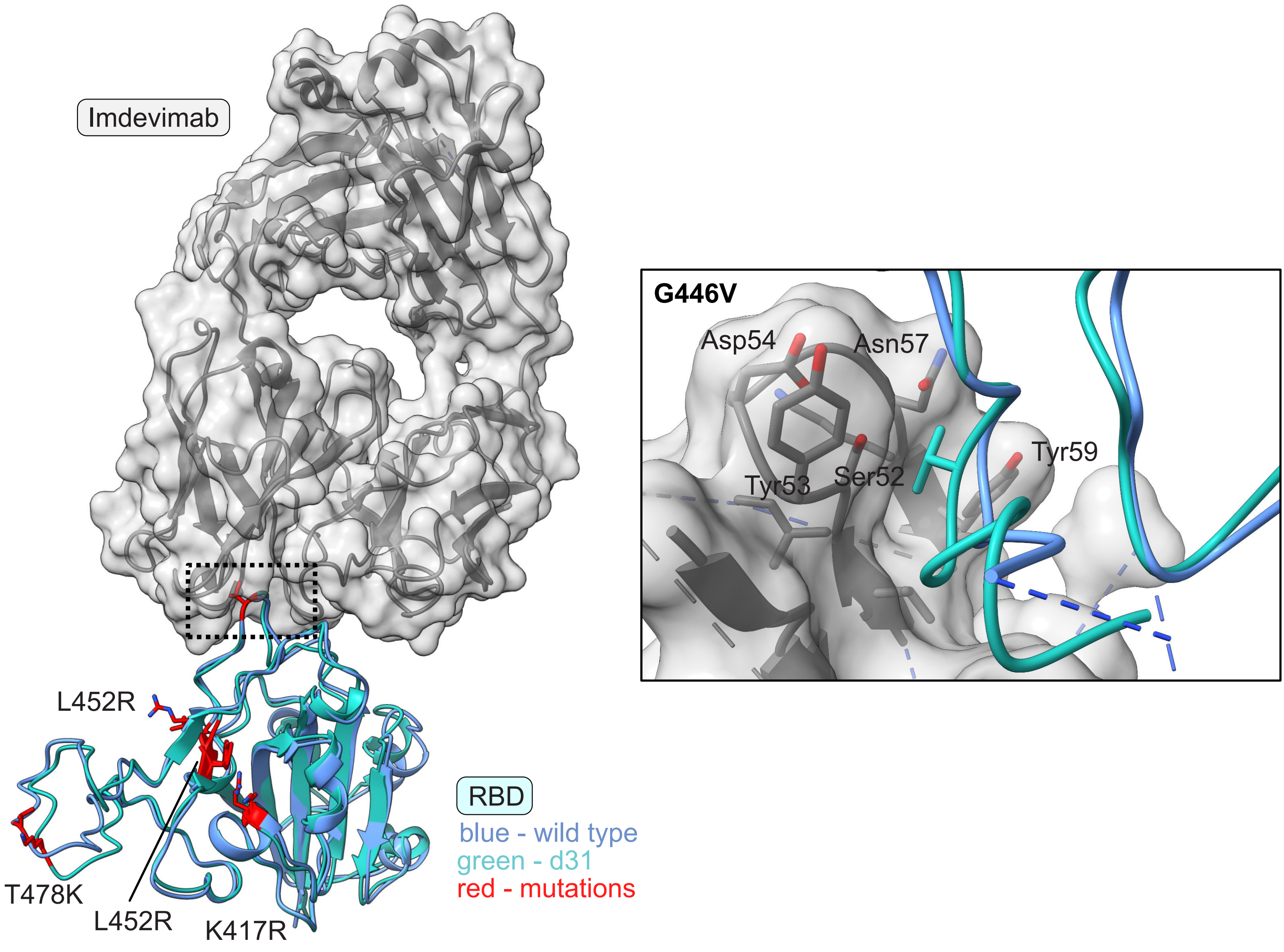

Supplementary figure 4. In depth overview of the S RBD Imdevimab interaction. 3D presentation of the RBD of the SARS-CoV-2 spike protein (PDB accession number: 6xdg, blue) and the d31 isolate RBD predicted by AlphaFold 2 (green) bound to the Fab fragments of Imdevimab. RBD mutations compared to Wuhan-Hu-1 (NC_045512.2) are marked in red. The dotted square marks the location of the G446V mutation which are enlarged on the right.

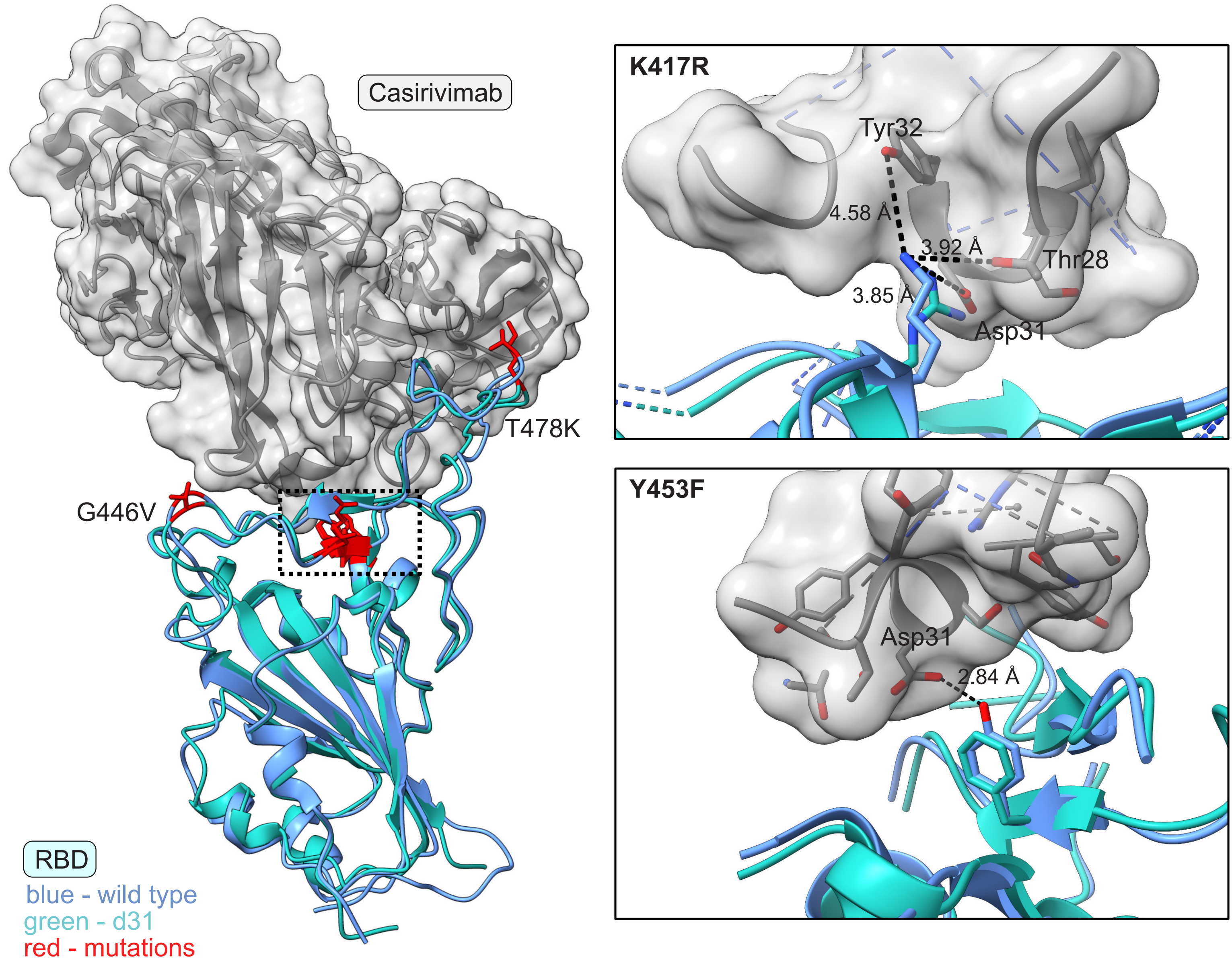

Supplementary figure 5. In depth overview of the S RBD Casirivimab interaction. 3D presentation of the RBD of the SARS-CoV-2 spike protein (PDB accession number: 6xdg, blue) and the d31 RBD predicted by AlphaFold 2 (green) bound to the Fab fragments of Casirivimab. RBD mutations compared to Wuhan-Hu-1 (NC_045512.2) are marked in red. The dotted square marks the location of the K417R and Y453F mutations which is enlarged on the right.

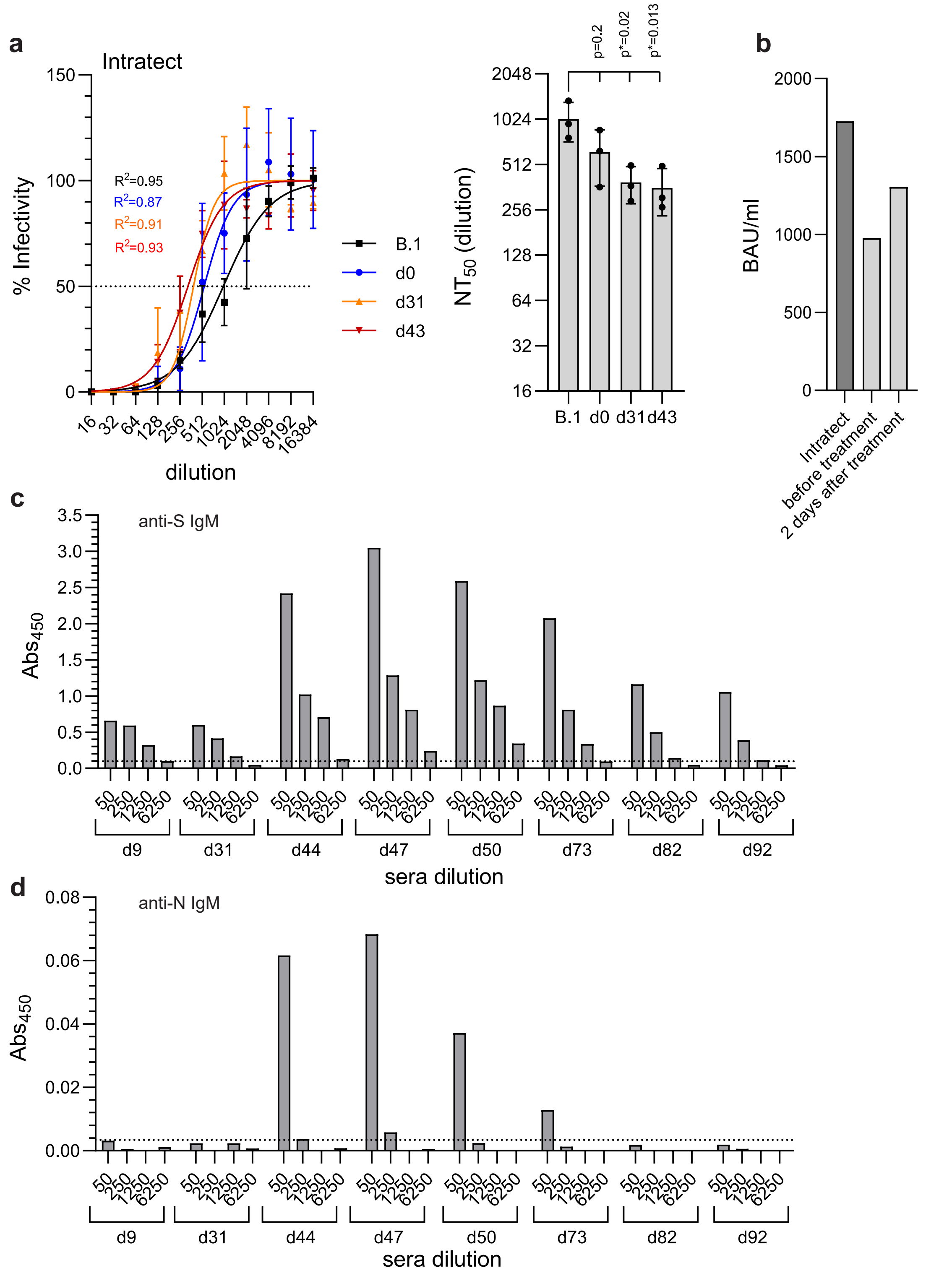

Supplementary figure 6. Presence of SARS-CoV-2 specific antibodies in intravenous total IgG preparations. (a) Neutralizing capacity of the patients' sera. Serial 2-fold dilutions of the Intratect IVIG that patient 1 received after she cleared the SARS-CoV-2 infection were incubated with 100 pfu of the B.1 isolate or the three patient isolates (d0, d31, d43) and analysed by plaque assay. Plotted are the curve fits (mean ±SD) of the serial dilutions (n=3 independent experiments) (left) and NT_{50} values of each experiment (right). The goodness of fit (R^2) is indicated at the respective curves. For the NT_{50} values the geometric mean and geometric standard deviation are shown. The dotted line marks 50 % infectivity. Statistics were performed on log-transformed values with a one-way ANOVA (Dunnett's multiple comparison test, *$p<0.05$). (b) Anti-S IgG ELISA of the Intratect IVIG batch and of patient 1 sera directly before and two days after IVIG treatment. Detection of SARS-CoV-2 anti-S (c) or anti-N (d) specific IgM by ELISA. Shown is the mean of two technical duplicates. Horizontal lines mark the detection limits. Source data are provided in the source data file.

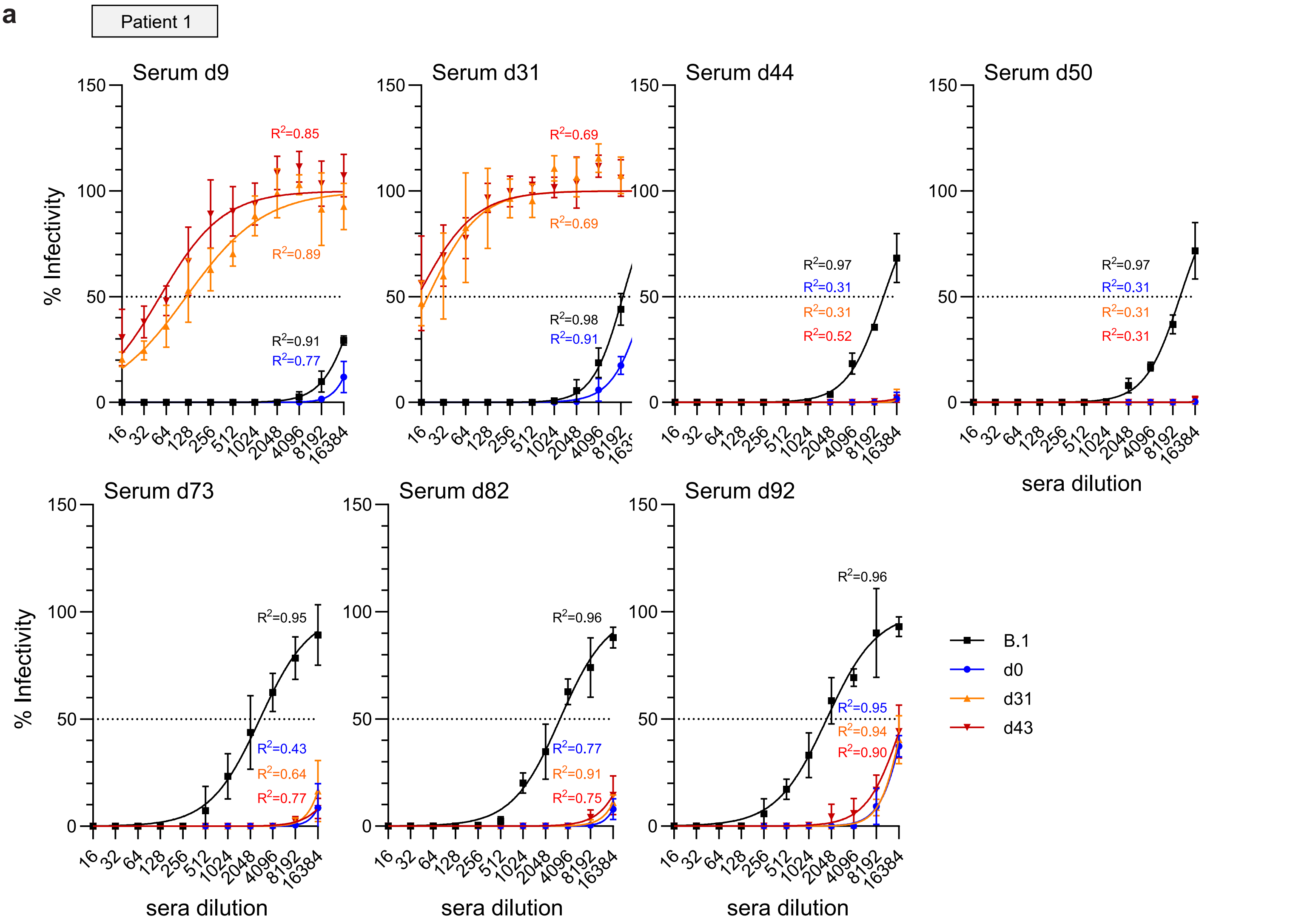

b

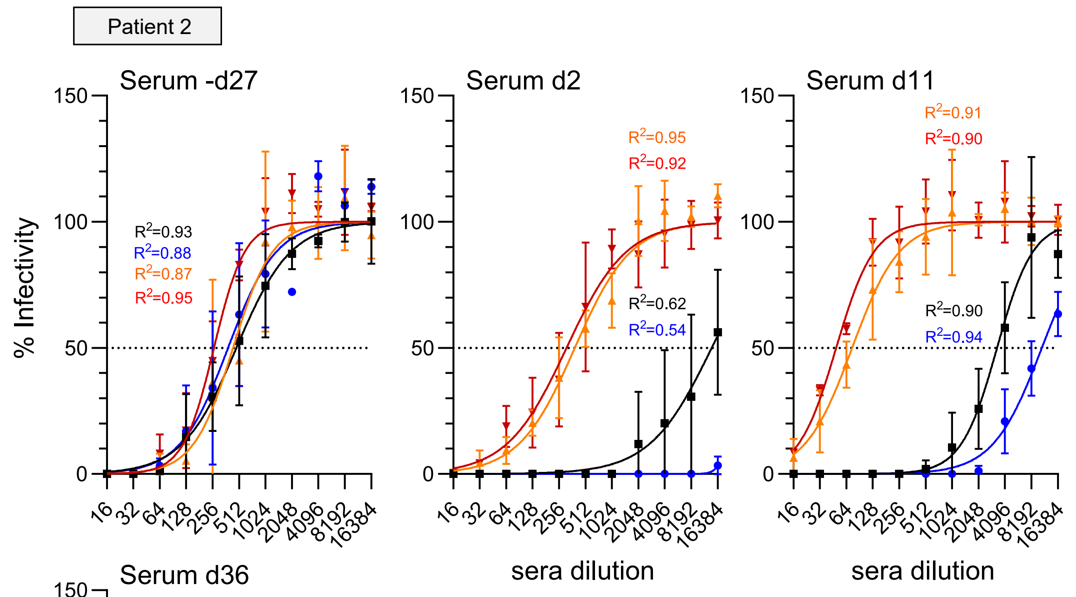

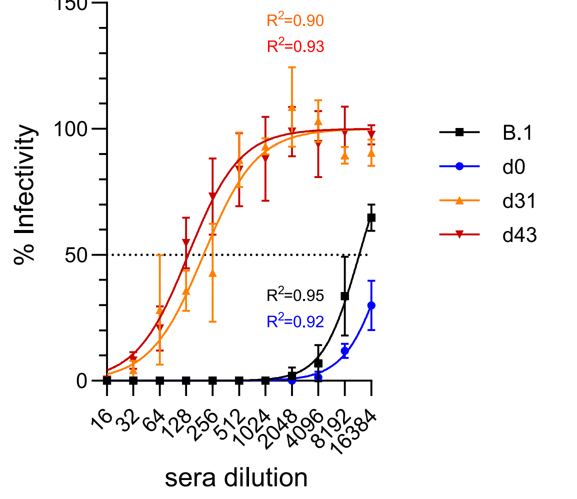

Supplementary figure 7. Curve fits for the neutralizing capacity of the sera of patient 1 and 2. Serial 2-fold dilutions of the sera of (a) patient 1 and (b) patient 2 were incubated with 100 pfu of the B.1 isolate or the three patient isolates (d0, d31, d43) and analysed by plaque assay. Plotted are the curve fits (mean ±SD) of the serial dilutions (n=3 independent experiments). The goodness of fit (R^2) is indicated at the respective curves. The dotted line marks 50 % infectivity. Source data are provided in the source data file.

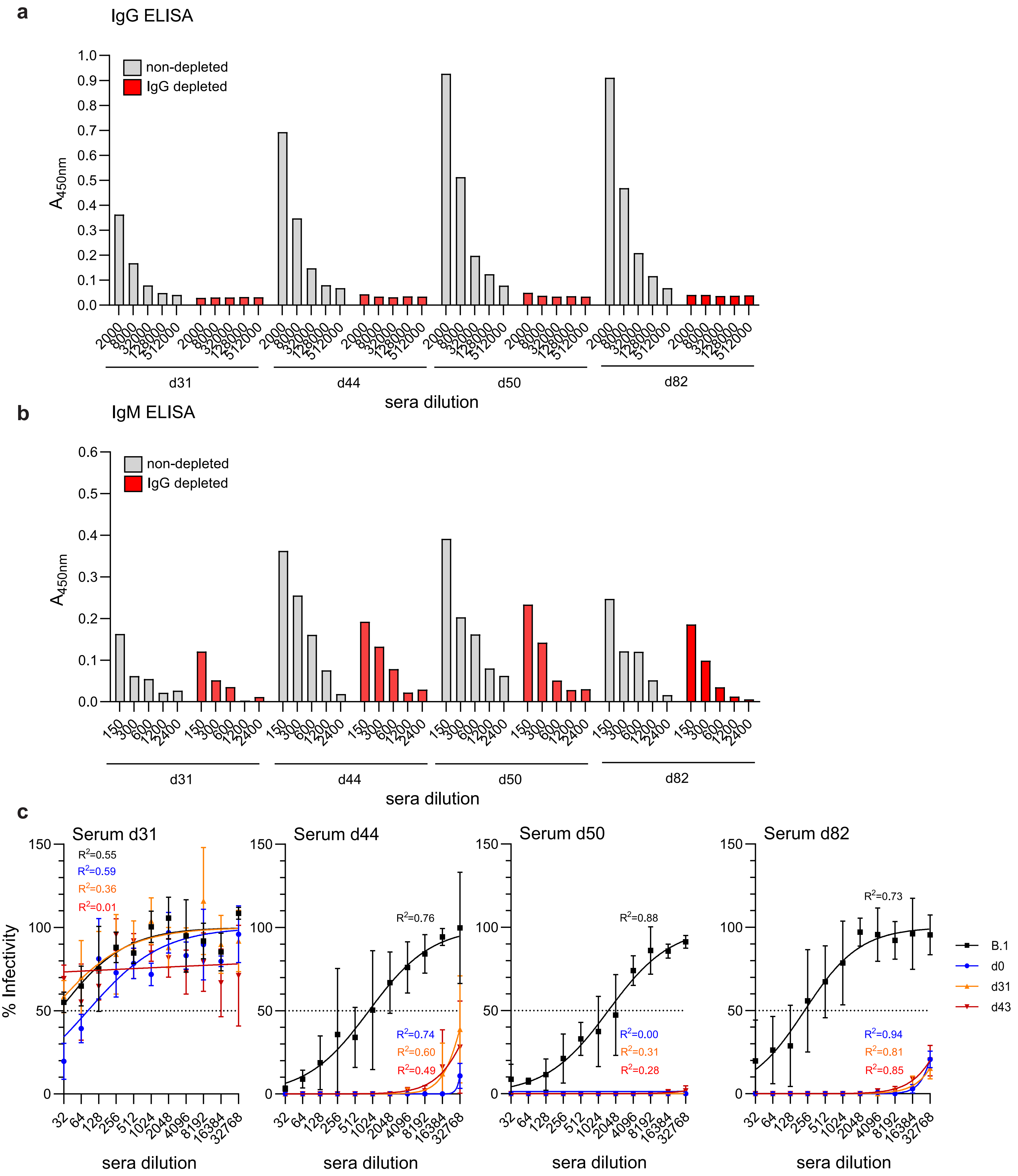

Supplementary figure 8. Depletion of IgG in sera of patient 1. IgG was depleted from sera of patient 1 at d31, d44, d50 and d82 post first positive qPCR result after incubation with protein G sepharose for 1 h at 4°C. After centrifugation supernatant was harvested and serial dilutions were assessed for (a) IgG and (b) IgM concentrations by ELISA. Shown is the mean of two technical duplicates. (c) Serial 2-fold dilutions of the IgG depleted sera of patient 1 were incubated with 100 pfu of the B.1 isolate or the three patient isolates (d0, d31, d43) and analysed by plaque assay. Plotted are the curve fits (mean ±SD) of the serial dilutions (n=3 independent experiments). The goodness of fit (R^2) is indicated at the respective curves. The dotted line marks 50 % infectivity. Source data are provided in the source data file.

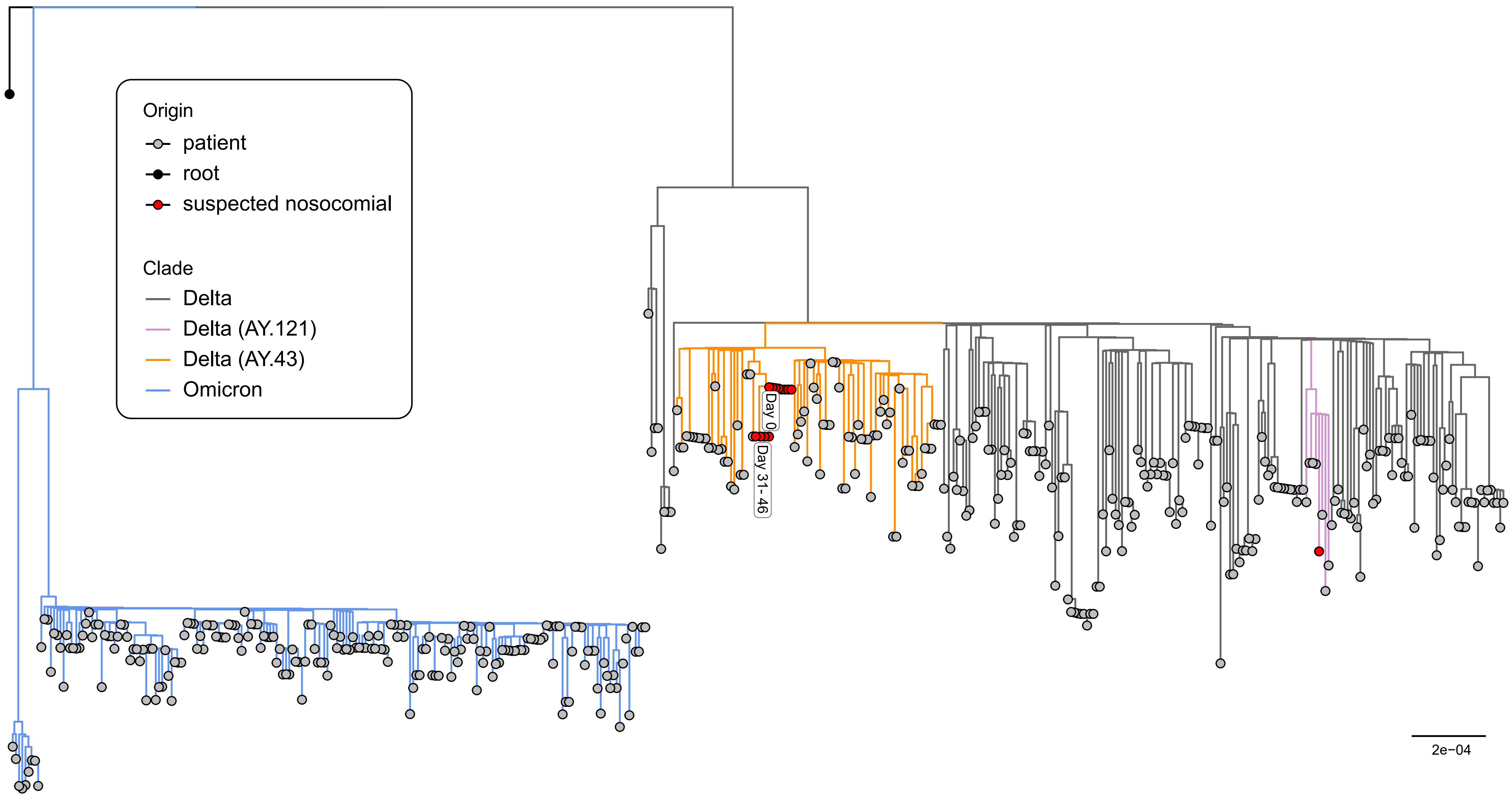

Supplementary figure 9. Phylogenetic tree of all patients at the University Medical centre Freiburg during the persistent SARS-CoV-2 infection of patient 1. The maximum-likelihood phylogenetic tree was constructed with IQ-Tree (1000 bootstrap replicates, GTR+F+R2) and rooted to the Wuhan-Hu-1 reference sequence (NC_045512). The tree was visualized with the R ggtree package. Lineages were assessed with pangolin v0.6 (data v1.8). Bar indicates substitutions per site.

Case	Date post index case	Nosocomial transmission	Direct contact	Ward	Vaccinated	Severity	Age	Ct value	Gender	Sequencing	Lineage
A	0	no	none	1	no	asymptomatic	80	18	male	successful	AY.43
B	3	yes	none	1	yes	mild	87	21	male	successful	AY.122.1
C	5	yes	B	1	yes	severe	60	24	male	unsuccessful	
D	4	yes	none	1	yes	severe	64	16	male	successful	AY.43
E	5	yes	D	1	no	mild	36	28	male	successful	AY.43
F	6	yes	D	1	yes	mild	63	20	male	successful	AY.43
G	7	yes	D	1	yes	mild	52	20	male	successful	AY.43
H	11	yes	none	2	yes	severe	70	14	female	successful	AY.43
I	12	suspected	none	3	yes	mild	61	20	female	successful	AY.43
J	14	suspected	none	3	no	severe (fatal)	52	22	female	successful	AY.43

Supplementary table 1. Relevant information for the nosocomial cases.
