# Supplementary material for: Total escape of SARS-CoV-2 from dual monoclonal antibody therapy in an immunocompromised patient
Source: Nat Commun. 2023 Apr 10;14:1999. doi: 10.1038/s41467-023-37591-w (PMC10085998; doi:10.1038/s41467-023-37591-w)
Supplement: Supplementary file 3 — Description of Additional Supplementary Files [file 41467_2023_37591_MOESM3_ESM.pdf]

Description of Additional Supplementary Files

Supplementary Data 1: All GISAID accession numbers and associated metadata.
