# Supplementary material for: Total escape of SARS-CoV-2 from dual monoclonal antibody therapy in an immunocompromised patient
Source: Nat Commun. 2023 Apr 10;14:1999. doi: 10.1038/s41467-023-37591-w (PMC10085998; doi:10.1038/s41467-023-37591-w)
Supplement: Supplementary file 5 — Reporting Summary [file 41467_2023_37591_MOESM5_ESM.pdf]

nature portfolio

Corresponding author(s): Jonas Fuchs and Marcus Panning

Last updated by author(s): Mar 16, 2023

Reporting Summary

Nature Portfolio wishes to improve the reproducibility of the work that we publish. This form provides structure for consistency and transparency in reporting. For further information on Nature Portfolio policies, see our Editorial Policies and the Editorial Policy Checklist.

Statistics

For all statistical analyses, confirm that the following items are present in the figure legend, table legend, main text, or Methods section.

n/a	Confirmed	
☐	☒	The exact sample size (*n*) for each experimental group/condition, given as a discrete number and unit of measurement
☐	☒	A statement on whether measurements were taken from distinct samples or whether the same sample was measured repeatedly
☐	☒	The statistical test(s) used AND whether they are one- or two-sided<br>*Only common tests should be described solely by name; describe more complex techniques in the Methods section.*
☒	☐	A description of all covariates tested
☒	☐	A description of any assumptions or corrections, such as tests of normality and adjustment for multiple comparisons
☐	☒	A full description of the statistical parameters including central tendency (e.g. means) or other basic estimates (e.g. regression coefficient) AND variation (e.g. standard deviation) or associated estimates of uncertainty (e.g. confidence intervals)
☐	☒	For null hypothesis testing, the test statistic (e.g. *F*, *t*, *r*) with confidence intervals, effect sizes, degrees of freedom and *P* value noted<br>*Give P values as exact values whenever suitable.*
☒	☐	For Bayesian analysis, information on the choice of priors and Markov chain Monte Carlo settings
☒	☐	For hierarchical and complex designs, identification of the appropriate level for tests and full reporting of outcomes
☒	☐	Estimates of effect sizes (e.g. Cohen's *d*, Pearson's *r*), indicating how they were calculated

Our web collection on statistics for biologists contains articles on many of the points above.

Software and code

Policy information about availability of computer code

Data collection	No Software was used.
Data analysis	Statistical analysis: GraphPad Prism (v.8.4.2) and R (v.4.0.2) using Rstudio ("Prairie Trillium")<br>Variant frequency visualization: github.com/jonas-fuchs/SARS-CoV-2-analyses (v.1.1, https://zenodo.org/badge/latestdoi/336032336).<br>Protein structure visualization: UCSF ChimeraX version: 1.1 (2020-09-09).<br>Analysis of NGS data: SARS-CoV-2 NGS data was analyzed on the cloud computing bioinformatic platform Galaxy (usegalaxy.eu, covid19.galaxyproject.org/artic/) using the following pipline: fastqs were preprocessed with fastp (v.0.20.1) and mapped using BWA-MEM (v.0.7.17), ARTIC primer sequences were trimmed using ivar trim (v1.9), SNPs and INDELs were called with lofreq (v2.1.5) and annotated with snpeff (v.4.3.1). Consensus sequences were generated with bcftools (v.1.10).<br>Phylogenetic tree: MAFFT (v.7.45), IQ-TREE multicore version (v2.1.2)<br>Pangolin assignment: pangolin (v0.6)<br>Pylogenetic tree visualization: R packages ggtree v2.2.4, treeio (v1.12.0) and ggplot2 (v3.3.3)<br>ColadFold: https://github.com/sokrypton/ColabFold (v1.2.0)

For manuscripts utilizing custom algorithms or software that are central to the research but not yet described in published literature, software must be made available to editors and reviewers. We strongly encourage code deposition in a community repository (e.g. GitHub). See the Nature Portfolio guidelines for submitting code & software for further information.

Data

Policy information about availability of data

All manuscripts must include a data availability statement. This statement should provide the following information, where applicable:

- Accession codes, unique identifiers, or web links for publicly available datasets
- A description of any restrictions on data availability
- For clinical datasets or third party data, please ensure that the statement adheres to our policy

All necessary data and information are given in the paper. Source data are provided with this paper. The sequence data were submitted to the GISAID database and are publicly available (Supplementary Data 1). The GISAID accession numbers of patient 1 are: EPI_ISL_7996735 (day 0), EPI_ISL_8898226 (day 31), EPI_ISL_8898236 (day 36), EPI_ISL_9324089 (day 40) and EPI_ISL_9324138 (day 43). Raw sequencing data and deduced consensus genomes have been also submitted to the European Nucleotide Archive under the study accession number: ERP139553 [https://www.ebi.ac.uk/ena/browser/view/PRJEB54706]. The pdb database accession numbers [https://www.rcsb.org/] that were used for structural analysis and visualization are: 7U0N (doi:10.2210/pdb7U0N/pdb, SARS-CoV-2 S binding ACE2) and 6XDG (doi: 10.2210/pdb6XDG/pdb, REGN-COV binding SARS-CoV-2 S). For SARS-CoV-2 lineage assignment pangolin data v1.8 [https://github.com/cov-lineages/pangolin-data/releases/tag/v1.8] was used. The sequence used in this study as the SARS-CoV-2 reference genome has the GenBank [https://www.ncbi.nlm.nih.gov/genbank/] accession: NC_045512 (Wuhan-Hu-1).

Human research participants

Policy information about studies involving human research participants and Sex and Gender in Research.

Reporting on sex and gender	Sex or gender was not considered in the study design and no sex- or gender-based analyses have been performed.
Population characteristics	Two female (70 and 61 years old) immunosupressed kidney transplant patients.
Recruitment	Both patients were recruited due to their unique history of REGN-COV treatment upon COVID-19 diagnosis, their immunosuppression and the prolonged infection of one patient. This biases findings torwards these two specific clinical cases.
Ethics oversight	The project has been approved by the ethical committee of the Albert-Ludwigs-Universität, Freiburg, Germany. Written informed consent was obtained from all participants and the study was conducted according to federal guidelines, local ethics committee regulations (Albert-Ludwigs-Universität, Freiburg, Germany: No. F-2020-09-03-160428 and no. 322/20) and the Declaration of Helsinki (1975). All routine virological laboratory testing of patient specimens (virus isolation and next-generation sequencing) was performed in the Diagnostic Department of the Institute of Virology, University Medical Center, Freiburg (Local ethics committee no. 1001913).

Note that full information on the approval of the study protocol must also be provided in the manuscript.

Field-specific reporting

Please select the one below that is the best fit for your research. If you are not sure, read the appropriate sections before making your selection.

☒ Life sciences ☐ Behavioural & social sciences ☐ Ecological, evolutionary & environmental sciences

For a reference copy of the document with all sections, see nature.com/documents/nr-reporting-summary-flat.pdf

Life sciences study design

All studies must disclose on these points even when the disclosure is negative.

Sample size	The sample size of the K18-hACE2 transgenic mice was estimated on the basis of experience with other respiratory viruses to give statistical power while minimizing animal use.<br>For all other experiments no sample size calculation was performed. Sample sizes were chosen based in the minimum number of independent experiments that allow statistical evaluation (n=3).
Data exclusions	No data were excluded.
Replication	Data were reproduced at least as biological triplicates. Diagnostic assays (ELISAs, qPCR of respiratory samples) were done once using accredited assays due to the limited availability of the individual samples.
Randomization	Age- and sex-matched K18-hACE2 transgenic mice were randomly assigned to the experimental groups. For all other experiments no randomization was applied as no group allocation was carried out.
Blinding	No blinding was caried out. For mice experiments, only objective parameters (survival and weight) were included in the study design. All available data were used and therefore blinding would not have affected the experiments or the analyes. For all other experiments, blinding was not needed because no group allocation was carried out and no subjective paramenters were measured.

Reporting for specific materials, systems and methods

We require information from authors about some types of materials, experimental systems and methods used in many studies. Here, indicate whether each material, system or method listed is relevant to your study. If you are not sure if a list item applies to your research, read the appropriate section before selecting a response.

Materials & experimental systems

n/a	Involved in the study
☒	☐ Antibodies
☐	☒ Eukaryotic cell lines
☒	☐ Palaeontology and archaeology
☐	☒ Animals and other organisms
☒	☐ Clinical data
☒	☐ Dual use research of concern

Methods

n/a	Involved in the study
☒	☐ ChIP-seq
☒	☐ Flow cytometry
☒	☐ MRI-based neuroimaging

Eukaryotic cell lines

Policy information about cell lines and Sex and Gender in Research

Cell line source(s)	VeroE6 cells (ATCC CRL-1586) and), Calu-3 cells (ATCC-HTB-5), Expi293F cells (A14528, Gibco)
Authentication	None of the cell lines were authenticated
Mycoplasma contamination	all cell lines were tested monthly negative for mycoplasma
Commonly misidentified lines (See ICLAC register)	no commonly misidentified cell lines were used in the study

Animals and other research organisms

Policy information about studies involving animals; ARRIVE guidelines recommended for reporting animal research, and Sex and Gender in Research

Laboratory animals	Transgenic 034860-B6.Cg-Tg(K18-ACE2)2PrlmanJ mice (Winkler et al., Nat Immunol. 2020 Nov;21(11):1327-1335. doi: 10.1038/s41590-020-0778-2.<br>Epub 2020 Aug 24) were purchased from The Jackson Laboratory and bred locally. Hemizygous 8-12-week-old male animals were used.<br>Mice were housed at 14-hour light/10-hour dark cycles and temperatures of ~18-23°C with 40-60% humidity.
Wild animals	no wild animals were used in the study
Reporting on sex	No sex based analyses were performed as sex matched mice had been randomly assigned to experimental groups.
Field-collected samples	no field collected samples were used in the study
Ethics oversight	All animal studies were performed in accordance with the guidelines of the Federation for Laboratory Animal Science Associations and the National Animal Welfare Body. All experiments were in compliance with the German animal protection law and approved by the animal welfare committee of the Regierungspraesidium Freiburg (permit G-20/91).

Note that full information on the approval of the study protocol must also be provided in the manuscript.
